# Density-dependence and different dimensions of changing weather shape adult abundance patterns of common mosquito species (Diptera: Culicidae) in Bloomington, Indiana, USA

**DOI:** 10.1016/j.crpvbd.2025.100242

**Published:** 2025-01-07

**Authors:** Aidan Patrick Simons, Amanda Lenfestey, Luis Fernando Chaves

**Affiliations:** a Department of Environmental and Occupational Health, School of Public Health, Indiana University, Bloomington, IN, 47408, USA; b Department of Geography, Indiana University, Bloomington, IN, 47401, USA

**Keywords:** Relative humidity, Temperature, Precipitation, Schmalhausen’s law, Cross-correlation

## Abstract

Understanding the factors driving changes in mosquito abundance are key to quantify the risk they pose as vectors of pathogens. Here, to study the impacts of weather changes and density-dependent regulation on mosquito species abundance, we used season long weekly time series of *Aedes japonicus* (Theobald), *Aedes triseriatus* (Say), *Aedes vexans* (Meigen), *Anopheles punctipennis* (Say), C*oquillettidia perturbans* (Walker), and *Culex pipiens* L., common mosquito species in the Bloomington, IN, USA, area. We use the forced Ricker model to estimate population growth and density-dependence parameters, as well as the forcing by weather variables. We found that weather factors important for the population dynamics of these species were different. We found that *Cx. pipiens* population dynamics was not associated with any weather variables, while *Ae. japonicus*, *Ae. triseriatus* and *Cq. perturbans* were forced by relative humidity, *Ae. vexans* by SD of rainfall, and *An. punctipennis* by the kurtosis of temperature. These results illustrate the diversity of ways in which mosquitoes can respond to changing weather patterns and highlight the need for a more nuanced understanding of how mosquitoes respond to climate change by coupling field studies with mathematical modeling.

## Highlights

• We use the Ricker model to study density dependence and weather forcing in common Midwest USA mosquito species.
• We consider rainfall, relative humidity and temperature as weather variables.
• We consider the forcing impact of average and higher order moments (SD and kurtosis) of those weather variables.
• Three species were sensitive to relative humidity, one to the SD of rainfall and one to temperature kurtosis.
• Results show the ubiquity of density dependence in mosquitoes with different responses to changing weather patterns.

## Graphical abstract

**Figure:**
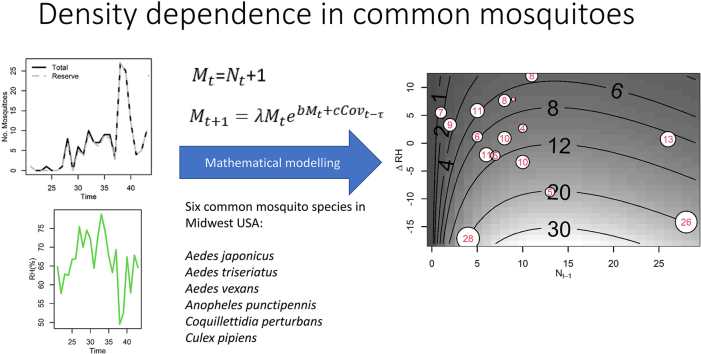

## 1 Introduction

Mosquitoes (Diptera: Culicidae) are insects that can transmit pathogens, posing significant health risks to humans, wild and domestic animals on a global scale. Understanding the impact of weather variables on the population dynamics of mosquitoes is crucial, to both understand the risk of pathogen transmission, and for controlling mosquito populations and reducing bite nuisance (Dietz et al., 1974; Chaves and Koenraadt, 2010; Chaves, 2017a). In insect ecology research, often the impacts of weather factors on insect abundance tend to focus on the role of single variables. In mosquitoes and other insects this has been illustrated by several studies that have highlighted the role of specific weather factors on mosquito population dynamics: temperature (Uvarov, 1931; Ewing et al., 2016); precipitation (Day and Curtis, 1989, 1994; Chase and Knight, 2003); or relative humidity (Thomson, 1938; Brown et al., 2023).

However, it is important to consider that the impacts of these variables can be very transient or depend on the larger ecological context where mosquitoes live (Juliano, 2009; Ruiz et al., 2010; Chaves et al., 2012, 2014; Shand et al., 2016; Poh et al., 2019; Brown et al., 2023). In addition, it is becoming more clear that we need to look at weather patterns beyond their means, as organisms respond not only to mean environments but to how widely and how often these environments fluctuate. This variability is described by the higher order moments of the statistical distribution of climatic variables, and biologically is relevant for living organisms, that experience the environment as a continuum. This is described by Schmalhausen’s law, the biological principle stating that organisms are more sensitive to small changes in some dimensions of their niche when stressed along other dimensions (Lewontin and Levins, 2000; Chaves, 2017a). For example, our previous work has shown how mosquito species are often more sensitive to changes in the variance or kurtosis of weather factors than their means (Chaves et al., 2012, 2015; Hoshi et al., 2017; Chaves and Moji, 2018). Similarly, mosquito ecology research has also shown that it is important to understand what is the interplay of weather fluctuations with density-dependence, the latter defined as the negative feedback that keeps mosquito and other populations regulated as function of their own abundance, where, in general, increases in abundance reduce the growth rate of populations (Turchin, 2003; Ranta et al., 2006; Royama, 2021). Density dependence is a pattern that has been described in several mosquito species using mathematical models (Yang et al., 2008a, 2008b; Hoshi et al., 2014). In this paper we seek to understand the impact that relative humidity, rainfall, temperature and their higher order moments, together with density dependence have on shaping the abundance of six common mosquito species in Bloomington, Indiana, USA (Khan et al., 2024): *Aedes japonicus* (Theobald), *Aedes triseriatus* (Say), *Aedes vexans* (Meigen), *Anopheles punctipennis* (Say), C*oquillettidia perturbans* (Walker), and *Culex pipiens* L.

The similarities and differences between these mosquito species, which are common across most of Midwest USA, the region where Bloomington is located, can enhance our understanding of how relative humidity, temperature, and precipitation affect mosquito abundance. First, the inclusion of both native, e.g. *Ae. triseriatus*, and invasive species, e.g. *Ae. japonicus*, allows a more comprehensive understanding of how changing environments might favor (or control) mosquito species invasion. Similarly, the inclusion of both univoltine species that have one generation per year like *Cq. perturbans*, and multivoltine species that have multiple generations per year like all the other species in this study, provides insight on how different reproductive habits are affected by weather factors (Numata and Shintani, 2023). Beyond these similarities and differences, our selected species represent a diversity of bionomics and natural histories, whose main characteristics we describe next, especially as they are suggestive of different response to weather changes.

*Aedes japonicus*, commonly known as the Asian Bush mosquito, is native to eastern Asia. *Aedes japonicus*, is an invasive species in North America and Europe, resistant to colder temperatures and readily oviposits in both man-made containers and tree holes (Kaufman and Fonseca, 2014; Medlock et al., 2015). *Aedes japonicus* has rapidly expanded in the USA while native species like *Ae. triseriatus* are decreasing, suggesting that *Ae. japonicus* is displacing native species (Kaufman and Fonseca, 2014). Prior studies in Japan have suggested that *Ae. japonicus* larvae are sensitive to changes in the standard deviation (SD) and kurtosis of water temperature in the larval habitats where they inhabit (Chaves and Moji, 2018), while adults are influenced by air temperature (Chaves, 2016). *Aedes japonicus* is medically important as it is a proven vector of Japanese encephalitis virus (Kitaoka et al., 1950; Takashima et al., 1988; Takashima and Rosen, 1989), La Crosse encephalitis virus (Harris et al., 2015; Westby et al., 2015), and it is vectorially competent to transmit West Nile virus (Sardelis and Turell, 2001), Eastern equine encephalitis virus (Sardelis et al., 2002) and St. Louis encephalitis virus (Sardelis et al., 2003).

Native to eastern and central North America, *Aedes triseriatus* is also known as the eastern tree-hole mosquito. *Aedes triseriatus* breeds in tree holes from deciduous trees and man-made containers such as flowerpots, birdbaths, and tires (Beier et al., 1983; Barker et al., 2003a, 2003b). Larvae are often more abundant in tire yards than in wooded environments due to the tires’ ability to hold water for prolonged periods of time (Nasci, 1988). The response to changing environments is sensitive in the egg stage, oviposition seems to follow rainfall (Kitron et al., 1989; Szumlas et al., 1996; Barker et al., 2003b), and to more commonly occur in forested areas, when compared with more urbanized landscapes (Barker et al., 2003a, 2003b). However, larvae and pupae are commonly found in tires, with higher larval abundance and adult productivity in shaded tires (Beier et al., 1983; Haramis, 1984; Nasci, 1988). Eggs are also a key stage for the overwintering of this mosquito species, where both photoperiods with short light periods (8 h) or low temperatures (≥ 10 °C) with long periods of daylight (16 h) can induce egg diapause, which is broken as eggs experience high temperatures (≥ 21 °C) for at least 13 days (Shroyer and Craig, 1980, 1983). However, larvae also diapause in response to low temperatures even when light in the photoperiod is long, with latitudinal patterns suggesting that egg diapause might be more common in northern/temperature populations, when compared with subtropical ones (Sinsko and Craig, 1981; Sims, 1982). Adult *Ae. triseriatus* production from pupae in tree holes also seems to follow rainfall, with the overall productivity of adults reduced during dry periods, and a variable phenology where adult production can start in early or late May and end by early or late September in temperate populations (Durso and Craig Jr, 1980; Sinsko and Craig, 1979, 1981). However, adults can be active up to late October or November in subtropical regions (Szumlas et al., 1996). Like oviposition, adult densities tend to be higher in forested areas when compared with other landscapes (Haramis and Foster, 1983; Walker et al., 1987; Pumpuni and Walker, 1989; Irby and Apperson, 1992). *Aedes triseriatus* has been recognized as the most important vector of La Crosse encephalitis virus in Midwest USA (Day et al., 2023).

*Aedes vexans*, known commonly as the inland flood water mosquito, can be found in North America, Europe, Asia, Australia, and Africa (Johansen et al., 2005). *Aedes vexans* is a highly adaptable species that lays eggs in temporary pools of water formed after rainfall (Chuang et al., 2011a; Li et al., 2023). *Aedes vexans* abundance has been positively associated with relative humidity (Platt et al., 1958) and also with rainfall, from one week to one month, temperatures over 12 °C, but with a negative association with maximum temperatures during the summer (Chuang et al., 2011a, 2012a; Ripoche et al., 2019). In different contexts, *Ae. vexans* has been negatively associated with precipitation, and positively with relative humidity (Baril et al., 2023). *Aedes vexans* is a mosquito vectorially competent to transmit Zika virus (Gendernalik et al., 2017) and West Nile virus (Turell et al., 2001).

*Anopheles punctipennis*, also known as the woodland malaria mosquito, is native to North America and is commonly found in swamps and nearby forested areas (Irby and Apperson, 1992; Lee et al., 2024). This species has the capacity to transmit *Plasmodium* spp. parasites causing malaria in humans; its role as a vector in the USA is less important compared to tropical regions (Robert et al., 2005). White-tailed deer are the primary blood source hosts to *An. punctipennis*, making this mosquito species an effective bridge vector between humans and deer in the transmission of Eastern equine encephalitis virus (Molaei et al., 2009). Rainfall is positively associated with its abundance in Midwest USA (Lee et al., 2024).

*Coquillettidia perturbans*, also known as the cattail mosquito, is native to eastern and central North America. *Coquillettidia perturbans* utilizes an anal siphon to attach to stems of aquatic plants such as cattails, giving them a constant source of oxygen until they emerge as adults (Bidlingmayer, 1968; Slaff and Haefner, 1985; Morris et al., 1990). Larvae are associated with aquatic plants, including arrow arum, *Peltandra* spp. (Bidlingmayer, 1968; Morris et al., 1990), cattail, *Typha* spp. (Batzer and Sjogren, 1986), sedge, *Carex* spp. (Callahan and Morris, 1987; Morris et al., 1990), water lettuce, *Pistia stratiotes*, water hyacinth, *Eichhornia crassipes* (Slaff and Haefner, 1985); maidencane, *Panicum hemitomon*, torpedograss, *Panicum repens*, pickerelweed, *Pontederia cordata*, bladderworth, *Utricularia* spp., among other species (Callahan and Morris, 1987). It has been suggested that beyond the association with these aquatic plants *Cq. perturbans* and related species select larval habitats in water bodies with low dissolved oxygen concentrations, pH around 5, and large amounts of detritus (Bidlingmayer, 1968; Batzer and Sjogren, 1986; Callahan and Morris, 1987; Morris et al., 1990). In temperate North America adult *Cq. perturbans* mosquitoes tend to have a unique monotonic peak in abundance (Lewis and Bennett, 1980; Allan et al., 1981; Batzer and Ranta, 1994; Olds et al., 1989), but in more subtropical environments it has been argued the possibility that multiple peaks represent multiple generations (Lounibos and Escher, 1983; Slaff and Haefner, 1985; Batzer et al., 2020). Regarding the association with weather variables, adults where more abundant at lower temperatures during the summer when sampled with aerial net sweeps (Lewis and Bennett, 1980), and mosquitoes trapped with CO_2_ baited CDC traps increase with minimum temperature and relative humidity (Baril et al., 2023) and larval abundance was negatively correlated with temperature, probably reflecting that this species overwinters as third- and fourth-instar larvae (Lounibos and Escher, 1983). *Coquillettidia perturbans* can transmit the viruses that cause Eastern and Western equine encephalitis (Nasci et al., 1996; Sherwood et al., 2020).

*Culex pipiens*, the northern house mosquito, is also a species of medical importance. This species is globally distributed and is the primary vector of West Nile virus in North America (Rochlin et al., 2019), but also of several species of filarial worms (Sasa, 1965). *Culex pipiens* oviposits in both manmade objects and tree holes (Zea Iriarte et al., 1991; Tsuda et al., 1994), catch basins (Munstermann and Craig, 1977), and polluted lentic water bodies (Britton, 1914; Mogi and Okazawa, 1990). Several studies have looked at the impacts of weather on its population dynamics. In general, it has been observed that *Cx. pipiens* abundance is dependent on having an adequate number of degree days, with abundance increasing with warmer temperatures (Hayes, 1975; Hayes and Hsi, 1975; Hayes and Downs, 1980; Shand et al., 2016; Ripoche et al., 2019). *Culex pipiens* adults also enter reproductive diapause following shorter daylengths and lower temperatures, which influence its seasonality that tends to peak during the summer in North America (Spielman, 2001; Barker et al., 2010). Low precipitation and high mean daily temperature were associated with high larval abundance (Gardner et al., 2012), so was high ammonia and nitrates in catch basins and the coverage of shrubs less than 1 m in height, tree density (Gardner et al., 2013). Meanwhile, adult abundance has been positively associated with minimum temperature, but negatively associated with summer precipitation and also with maximum temperature in July and August (Chuang et al., 2011b), a pattern observed for both larvae and adults around catch basins (Marini et al., 2021). Adult abundance has also been associated with more variable rainfall and temperature, as measured by their SD, and also with leptokurtic, or high kurtosis, rainfall (Chaves et al., 2011), and in general it has been observed that abundance is associated with higher order statistical moments of environmental variables, including temperature, rainfall but also vegetation indices (Poh et al., 2019).

Given the diversity of mosquito species responses to changing environments that we just described, it is our hypothesis that while all the mosquito species considered in this study will undergo density dependent population growth, their response to meteorological changes will be species and context specific. This means that we expect that climate and weather elements associated with mosquito species population dynamics will be different, both in terms of the elements that are most significantly associated with the dynamics and also regarding whether abundance is associated with mean values or higher order statistical moments of the weather variables we considered in this study.

## 2 Materials and methods

### 2.1 Study area

**Fig. 1:**
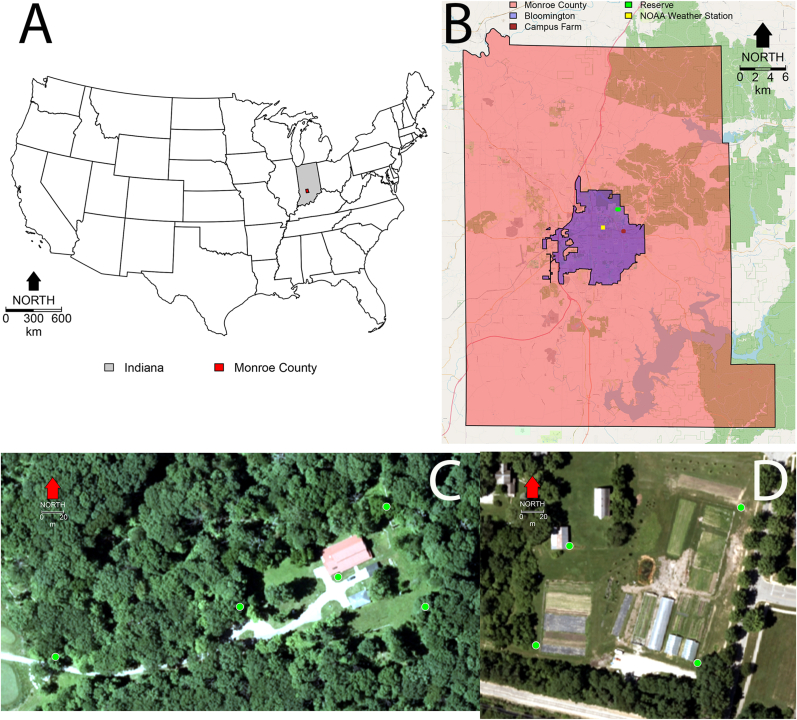
Study site. **A** Monroe County, Indiana. **B** Bloomington location within the limits of Monroe Co., IN, with indication of the location of the Bloomington weather station and the centroids of the farm site and reserve site. **C**, **D** Trap locations at the reserve (**C**) and the farm (**D**). For **B** we employed an image from OpenStreetMap (Brunsdon and Comber, 2015), while base images for figures **C** and **D** are courtesy of the US Department of Agriculture Farm Production and Conservation, Business Center, Geospatial Enterprise Operations and were obtained using the Google Earth Engine (Google, 2020).

Our research was carried out in Monroe County, south central Indiana (Fig. 1A), in the city of Bloomington (Fig. 1B). Our study was done at two sites: the Indiana University Research and Teaching Reserve (IURTR, Fig. 1C) and the Indiana University Campus Farm (IUCF, Fig. 1D). The IURTR is a forested area, abundant with wildlife and diverse plant life (Fig. 1C). In contrast, the IUCF is part of an urban environment, featuring nearby roads, buildings, land used for agriculture, and sparse vegetation (Fig. 1D).

### 2.2 Mosquito sampling

We sampled adult mosquitoes using BG-sentinel traps (Biogents AG, Regensburg, Germany) set in the CDC style at 1.2 m above the ground. Specific locations at the IURTR (5 traps) and IUCF (4 traps) can be seen, respectively, in Fig. 1C and D. Traps were deployed every Monday at 5:00 pm and collected the following Tuesday at 9:00 am weekly from May 23rd (epidemiological week 21) to November 14th, 2023 (epidemiological week 46). Each trap was baited with both a BG-Lure and BG-Pro LED lights. After collection, mosquitoes were killed by exposing them to −20 °C for 20 min and then adult mosquitoes were identified using a dissection microscope (S9E Stereo Zoom; Leica, Wetzlar, Germany) and taxonomic keys for adults of common mosquito species in Indiana (Siverly, 1972). To separate *Cx. pipiens* and *Cx. restuans* we employed a recently developed key based on morphological characters that show different ranges of variability in morphological characters and that allow the separation of these two species (Ferreira-de-Freitas et al., 2020). Further details about mosquito sampling have been previously reported by Khan et al. (2024).

### 2.3 Weather data

In this study, weather data for our study area was acquired from the Indiana University weather station (GHCND: USC00120784; coordinates: 39.17399°N, 86.52076°W, Fig. 1B). This station stores daily records of precipitation, maximum and minimum temperature. The data procurement was done through the Climate Data Online tool provided by the National Centers for Environmental Observation of NOAA (NOAA, 2024). Additionally, estimates for maximum and minimum relative humidity in Monroe County were obtained utilizing the climate engine (Huntington et al., 2017), which draws from Gridmet, a high-spatial resolution daily dataset of surface meteorological data across the contiguous USA (Abatzoglou, 2013). To derive a representative daily average, maximum and minimum daily values for both temperature and relative humidity were averaged. Weekly mean (sum for rainfall), standard deviation, and kurtosis for each weather variable were computed based on daily records, in order to test the prediction from Schmalhausen’s law that organisms are sensitive not only to average environments but also to their variability (Chaves, 2017a). We were unable to estimate the kurtosis of rainfall in weeks where the was no rainfall, as the kurtosis will be infinite (Ross, 2014), and we did not consider it further in our analysis.

### 2.4 Mathematical modelling and statistical analysis

For our analysis we employed the Ricker model

(1) $$N_{t+1}={\lambda N}_{t}e^{{bN}_{t}}$$

a simple two-parameter model that describes population growth during a one-time step, i.e., the change from $N_{t}$ to $N_{t+1}$, the parameters being one describing the population intrinsic growth rate (λ) and one for density dependence (*b*), which implies density dependence when its value is negative (*b* < 0). This model has been extensively studied, and it has been shown that when ln(*λ*) < 2, the population is stable, and fluctuations are in principle due to perturbations that can include the response of the population to environmental changes (Turchin, 2003; Mangel, 2006). To render feasible the analysis, given the presence of zeroes during the period where we collected the six species included in this analysis, we added one to the studied time series. The resulting time series, i.e.

*M_t_* = *N_t_* + 1, was used to fit the Ricker model:

(2) $$M_{t+1}={\lambda M}_{t}e^{bM_{t}}$$

assuming a Poisson distribution for $M_{t}$ and fitted using a maximum likelihood method we have described in detail elsewhere (Chaves et al., 2015; Hoshi et al., 2017). We also fitted a forced version of model (2) for *M*_*t*_:

(3) $$M_{t+1}={\lambda M}_{t}e^{bM_{t}+c{Cov}_{t-\tau }}$$

where *c* is a parameter describing the impact of weather covariates (*Cov*) with a time lag τ. For fitting the models, we removed the mean of the covariate time series, thus ensuring its mean value was zero, something that enables the interpretation of parameter *c* as the response to anomalies in the weather covariate (Chaves, 2017b; Chaves and Friberg, 2021). Covariates and time lags were chosen using a standard time series analysis technique where the cross-correlation function (CCF), a function showing the correlation of two variables at different time lags, in this case used to study the association of mosquito abundance ($N_{t}$) with each of the weather covariates, was estimated and inspected (Shumway and Stoffer, 2017).

The CCF analysis has the advantage of showing the association between two time series at different time lags, allowing to test if the association between weather variables and abundance is lagged. Covariates and time lags were chosen based on their significance (*P* < 0.05), and in the case that more than one lag was significant for the same covariate, we tested one at a time. All the resulting models were compared and the best models for each mosquito species were chosen based on the minimization of the Akaike information criterion, a metric that is used to minimize the number of parameters in a model while considering the data goodness-of-fit to the model as measured by the maximum likelihood that is optimized when parameters are estimated (Faraway, 2004). We estimated the 95% confidence limits for each of the parameters in the best model by profiling the likelihood (Bolker, 2008).

## 3 Results

### 3.1 Sampling and descriptive patterns

**Fig. 2:**
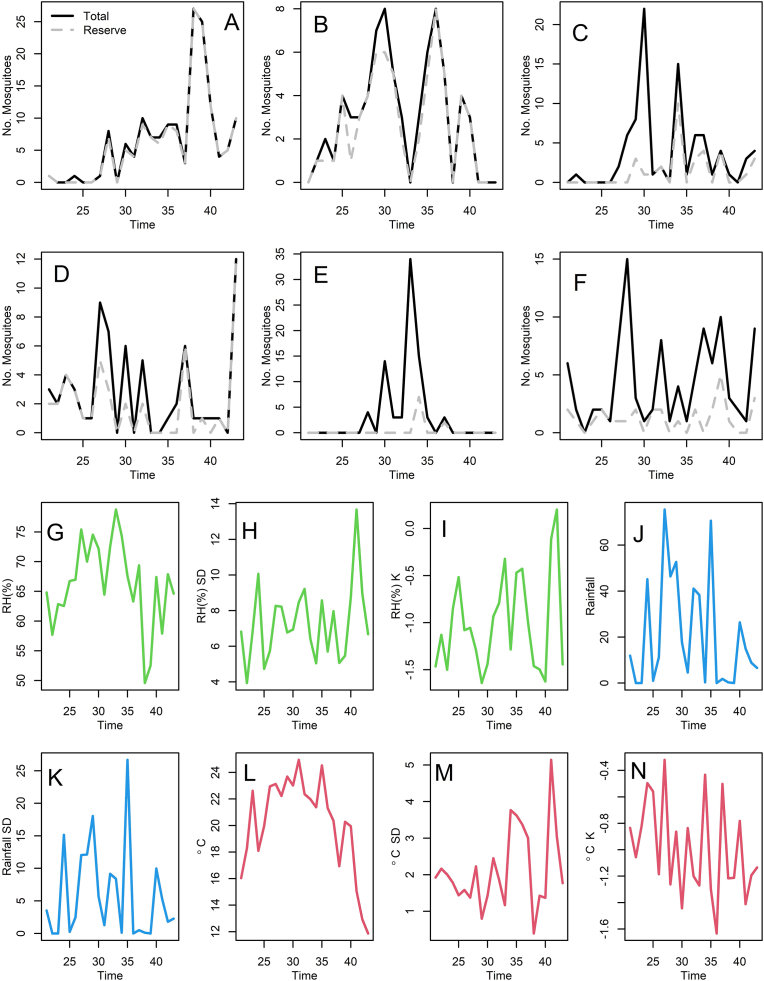
**A**-**F** Weekly time series. Abundance of *Aedes japonicus* (**A**), *Aedes triseriatus* (**B**), *Aedes vexans* (**C**), *Anopheles punctipennis* (**D**), *Coquillettidia perturbans* (**E**), and *Culex pipiens* (**F**). **G**-**N** Weather variables. **G** Average relative humidity. **H** SD of relative humidity. **I** Kurtosis of relative humidity. **J** Total rainfall. **K** SD of rainfall. **L** Average temperature. **M** SD of temperature. **N** Kurtosis of temperature. The grey dashed lines in panels **A** through **F** indicate the abundance at the reserve, while black solid lines indicate the weekly cumulative abundance for the farm and the reserve. RH was measured as % (panels **G**, **H** and **I**), rainfall in mm (panels **J** and **K**) and temperature in degrees Celsius (°C in panels **L**, **M** and **N**).

During our study we collected a total of 703 adult mosquitoes over a total sampling effort of 207 trap-nights, a sampling effort large enough to estimate the average weekly abundance of mosquito species during a season. Mosquitoes belonged to 19 species, and the six dominant species, *Ae. japonicus*, *Ae. triseriatus*, *Ae. vexans*, *An. punctipennis*, *Cq. perturbans* and *Cx. pipiens*, accounted for nearly 70% of the samples, totaling 491 mosquitoes. Further details about the full mosquito community have been presented elsewhere (Khan et al., 2024). In total, 146 *Ae. japonicus* specimens were collected, consisting of 46 males and 100 females. A total of 63 *Ae. triseriatus* specimens were collected, consisting of 58 females and 5 males. A total of 88 *Ae. vexans* specimens were collected, consisting of 66 females and 17 males. A total of 66 *An. punctipennis* specimens were collected, consisting of 42 females and 24 males. A total of 71 *Cq. perturbans* specimens were collected, consisting of 50 females and 21 males. A total of 57 *Cx. pipiens* specimens were collected, consisting of 24 females and 33 males. The average weekly adult abundance (mean ± SD), combining all traps and sex, of the mosquito species was as follows: *Ae. japonicus*: 6.08 ± 7.10; *Ae. triseriatus*: 2.73 ± 2.39; *Ae. vexans*: 3.67 ± 5.19; *An. punctipennis*: 2.75 ± 3.15; *Cq. perturbans*: 3.09 ± 7.27; and *Cx. pipiens*: 2.48 ± 2.80. Weekly abundance values ranged as follows: *Ae. japonicus*: 0–25; *Ae. triseriatus*: 0–8; *Ae. vexans*: 0–22; *An. punctipennis*: 0–12; *Cq. perturbans*: 0–34; and *Cx. pipiens*: 0–10. Next, we will describe the abundance changes for each mosquito species and the temporal evolution of the weather covariates during our study period (Fig. 2).

Referring to Fig. 2A, after a steady increase throughout the season, *Ae. japonicus* had its peak at 27 mosquitoes at week 38. *Aedes japonicus* was more abundant, and frequently found, in the reserve. *Aedes triseriatus* peaked at 8 mosquitoes at weeks 30 and 36, mostly at the reserve (Fig. 2B). Between week 20 and 31, most *Ae. vexans* mosquitoes were found at the farm with a peak of 23 mosquitoes at week 30 (Fig. 2C). After week 31, most *Ae. vexans* mosquitoes were found at the reserve with a peak of 15 mosquitoes at week 34. *Aedes vexans* had a season peak in the middle around weeks 28 and 35 with a steady increase before those weeks and a steady decrease after those weeks. *Anopheles punctipennis* mosquitoes were evenly split between the reserve and the farm between weeks 27 and 32 (Fig. 2D). *Anopheles punctipennis* had a relatively steady number of mosquitoes (6 mosquitoes) throughout the study season with a peak of 12 mosquitoes at week 45. *Coquillettidia perturbans* was only found between the weeks of 28 and 37 at the farm with a peak of 35 mosquitoes at week 33 with a steady increase before and a steady decrease after the peak (Fig. 2E). *Culex pipiens* were collected mostly evenly between the farm and the reserve with a steady stream of mosquitoes throughout the study season of around 5 mosquitoes (Fig. 2F). The peak of the season for *Cx. pipiens* was 15 mosquitoes at week 28. Relative humidity (%) hovered around 70 and had a peak of 79% at week 32 and a dip to 50% at week 37 (Fig. 2G). Relative humidity (%) standard deviation was steady around 7 and had a peak of 14 at week 40 (Fig. 2H). Relative humidity (%) kurtosis was around −1.0 with a peak of 0.1 at week 42 and two dips of −1.6 at weeks 29 and 40 (Fig. 2I). Rainfall hovered around 30 mm with two peaks of 78 mm at weeks 27 and 35 and two dips of 0 mm at weeks 36 and 39 (Fig. 2J). Rainfall standard deviation was steady around 7.5 with a peak of 26 at week 35 and two dips of 0 at weeks 36 and 39 (Fig. 2K). Temperature (°C) hovered around 21 until week 37 when the temperature started to steadily decrease and had a peak of 25 at week 32 and a dip of 12 at week 45 (Fig. 2L). Temperature (°C) standard deviation hovered around 2 but slowly increased over time with a peak of 5 at week 41 and a dip of 0 at week 38 (Fig. 2M). Temperature (°C) kurtosis was −1 with a slow decrease over time with a peak of −0.3 at week 26 and a dip of −1.6 at week 36 (Fig. 2N).

### 3.2 Mathematical modelling

For each mosquito species, we fitted the Ricker model to assess the impact of density dependence on their population dynamics. Parameter estimates for *λ* and *b* and their 95% confidence intervals (CI) are presented in Table 1. The intrinsic rate of population growth (*λ*) had a minimum of 2.150 (CI: 1.619–2.833) for *Ae. japonicus* and a maximum of 4.609 (CI: 3.123–6.674) for *An. punctipennis*. The density-dependent parameter *b* values had a maximum of −0.054 (CI: −0.034 to –0.075) for *Ae. japonicus* and a minimum of −0.338 (CI: −0.243 to –0.443) for *An. punctipennis*. Weather covariate selection is presented in Table 2. Table 3 shows parameter estimates *λ, b* and *c* and their 95% CIs considering the environmental covariates selected in Table 2. For *Cx. pipiens*, the best model selected did not include any environmental covariates (Table 2). To completely understand the relationships between species abundance and weather, taking lags into account is crucial. Lags farther out than 4 weeks were not used to fit the models for Table 3 because a mosquito’s life expectancy as adult is a month long (Clements and Paterson, 1981; Styer et al., 2007), and more than 4 weeks would imply a transgenerational impact on mosquito abundance (Chaves et al., 2014). We found that relative humidity was the weather covariate most significantly associated with: *Ae. japonicus* without lag and a negative association; *Ae. triseriatus* where it was associated with an increase in abundance two weeks after a period of high relative humidity; and *Cq. perturbans*, where it showed an increase in abundance immediately after a period of high humidity (0-week lag). SD and kurtosis of relative humidity were not statistically correlated for any species. Although total weekly rainfall was associated with three species, it was not selected because it was not the best-fit model for any of the mosquito species (Table 2). SD of total weekly rainfall correlated with three species, but it was only selected as the best-fit model for *Ae. vexans* (Table 2)*.* Average temperature was significant for *Ae. triseriatus*, but it was not selected because there the model with relative humidity as covariate was selected as best (Table 2). SD of average temperature was not selected for any species, although it was significantly associated with *Ae. japonicus* and *An. punctipennis* (Table 2). The best-fit model for *An. punctipennis* included the kurtosis of temperature as weather covariate (Table 2).

**Table 1:** Parameter estimates for the Ricker model fit to season-long weekly time series of common mosquito species in Bloomington, IN, USA, sampled from May to October of 2023. For the fitting, we employed a Poisson maximum likelihood described by Hoshi et al. (2017).

Species	Parameter	Estimate	2.5% CI	97.5% CI	SE	Z	Pr(z)
*Aedes japonicus*	$\hat{\lambda }$	2.150	1.619	2.833	0.306	7.016	2.29e-12∗
	$\hat{b}$	−0.054	−0.034	−0.075	0.010	−5.148	2.63e-07∗
*Aedes triseriatus*	$\hat{\lambda }$	2.366	1.358	3.979	0.648	3.652	0.00026∗
	$\hat{b}$	−0.150	−0.056	−0.246	0.048	−3.103	0.00192∗
*Aedes vexans*	$\hat{\lambda }$	3.736	2.689	5.147	0.618	6.043	1.52e-09∗
	$\hat{b}$	−0.169	−0.121	−0.215	0.024	−6.816	9.38e-12∗
*Anopheles punctipennis*	$\hat{\lambda }$	4.609	3.123	6.674	0.892	5.170	2.34e-07∗
	$\hat{b}$	−0.338	−0.243	−0.443	0.051	−6.659	2.76e-11∗
*Coquillettidia perturbans*	$\hat{\lambda }$	3.506	2.508	4.811	0.582	6.025	1.70e-09∗
	$\hat{b}$	−0.084	−0.061	−0.109	0.012	−6.946	3.77e-12∗
*Culex pipiens*	$\hat{\lambda }$	2.722	1.926	3.795	0.4712	5.786	7.22e-09∗
	$\hat{b}$	−0.147	−0.097	−0.201	0.026	−5.593	2.23e-08∗

*Abbreviations*: CI, confidence interval; SE, standard error; Z, score from a standard normal distribution; Pr(z), probability of the Z-score being different from a standard normal distribution with mean zero.

*∗* Statistically significant (*P* < 0.01).

**Table 2:** Lags of weather variables significantly correlated with mosquito abundance and model selection for the forced Ricker model.

Species	RH-Mean	RH-SD	RH-K	Rain-Total	Rain-SD	Temp-Mean	Temp-SD	Temp-K	No weather
*Aedes japonicus*	(0, –) **131.60**	NS	NS	NS	NS	NS	(4, +) NT	NS	162.47
*Aedes triseriatus*	(2, +) **82.75**	NS	NS	(2, +) 86.93	(1, +) 85.20	(0, +) 86.94	NS	NS	88.24
*Aedes vexans*	NS	NS	NS	(1, +) 119.32	(1, +) **118.07**	NS	NS	NS	130.54
*Anopheles punctipennis*	(5, –) NT	NS	NS	NS	(8, +) NT	NS	(2, +) 117.64	(3, +) **102.60**	121.82
*Coquillettidia perturbans*	(0, +) **91.71**	NS	NS	(6, +) NT	NS	NS	NS	NS	158.30
*Culex pipiens*	NS	NS	NS	NS	NS	NS	NS	NS	**129.69**

*Notes*: Each row indicates a common mosquito species in Bloomington, IN, USA, while each column is a weather variable, including relative humidity (RH), rainfall (Rain), temperature (Temp), including the mean, standard deviation (SD) and kurtosis (K). Values inside parentheses indicate the lag, in weeks, and the sign of the association, positive (+) or negative (–). Values outside parentheses indicate the Akaike information criterion (AIC) where the lowest value for each species is indicated in bold. Lags over 3 weeks where not tested (NT) and weather variables without significant associations are indicated by NS. “No weather” indicates a model without a climate covariate, i.e. the models whose parameters are presented in Table 1.

**Table 3:** Parameter estimates for the forced Ricker model fit to season-long weekly time series of common mosquito species in Bloomington, IN, USA, sampled from May to October 2023. For the fitting, we employed a Poisson maximum likelihood described by Hoshi et al. (2017).

Species	Weather covariate (lag in weeks)	Parameter	Estimate	2.5% CI	97.5% CI	SE	Z	Pr(z)
*Aedes japonicus*	Relative Humidity Mean (0)	$\hat{\lambda }$	2.398	1.825	3.121	0.328	7.316	2.55e-13∗
		$\hat{b}$	−0.074	−0.055	−0.095	0.010	−7.270	3.59e-13∗
		$\hat{c}$	−0.061	−0.080	−0.042	0.010	−6.261	3.83e-10∗
*Aedes triseriatus*	Relative Humidity Mean (2)	$\hat{\lambda }$	2.690	1.552	4.495	0.729	3.690	0.0002∗
		$\hat{b}$	−0.184	−0.088	−0.280	0.049	−3.760	0.00017∗
		$\hat{c}$	0.046	0.013	0.080	0.017	2.655	0.00792∗
*Aedes vexans*	Rainfall SD (1)	$\hat{\lambda }$	3.169	2.228	4.446	0.558	5.681	1.33e-08∗
		$\hat{b}$	−0.150	−0.105	−0.200	0.024	−6.175	6.63e-10∗
		$\hat{c}$	0.052	0.029	0.075	0.012	4.469	7.86e-06∗
*Anopheles punctipennis*	Temperature Kurtosis (3)	$\hat{\lambda }$	3.243	2.117	4.877	0.690	4.701	2.59e-06∗
		$\hat{b}$	−0.278	−0.187	−0.380	0.049	−5.669	1.44e-08∗
		$\hat{c}$	1.649	0.994	2.332	0.340	4.845	1.26e-06∗
*Coquillettidia perturbans*	Relative Humidity Mean (0)	$\hat{\lambda }$	2.134	1.435	3.065	0.411	5.185	2.16e-07∗
		$\hat{b}$	−0.068	−0.050	−0.089	0.010	−6.994	2.68e-12∗
		$\hat{c}$	0.194	0.144	0.249	0.027	7.247	4.26e-13∗

*Abbreviations*: CI, confidence interval; SE, standard error; Z, score from a standard normal distribution; Pr(z), probability of the Z-score being different from a standard normal distribution with mean zero.

*∗* Statistically significant (*P* < 0.01).

When considering the impact of weather variables on population dynamics (Table 3), the values for the intrinsic rate population growth (λ) had a minimum of 2.134 (CI: 1.435–3.065) for *Cq. perturbans* and a maximum of 3.243 (CI: 2.117–4.877) for *An. punctipennis*. The density-dependent parameter *b* values had a maximum of −0.068 (CI: −0.050 to –0.089) for *Cq. perturbans* and a minimum of −0.184 (CI: −0.088 to –0.280) for *Ae. triseriatus*. Parameter values for the impact of weather variables on abundance, *c*, had a negative value at −0.061 (CI: −0.080 to −0.042) for *Ae. japonicus* and when positive its values ranged from a minimum of 0.046 (CI: 0.013–0.080) for *Ae. triseriatus* and a maximum of 1.649 (CI: 0.994–2.332) for *An. punctipennis*. The value of *c* for *An. punctipennis* was an order of magnitude higher than the other species due to kurtosis of temperature values being low. For *Ae. japonicus* and *Ae. triseriatus* the parameters λ and −*b* increased their values when considering the weather covariates, while the opposite, λ and −*b* decreasing their values, happened for *Ae. vexans*, *An. punctipennis* and *Cq. perturbans* (Table 1, Table 3).

**Fig. 3:**
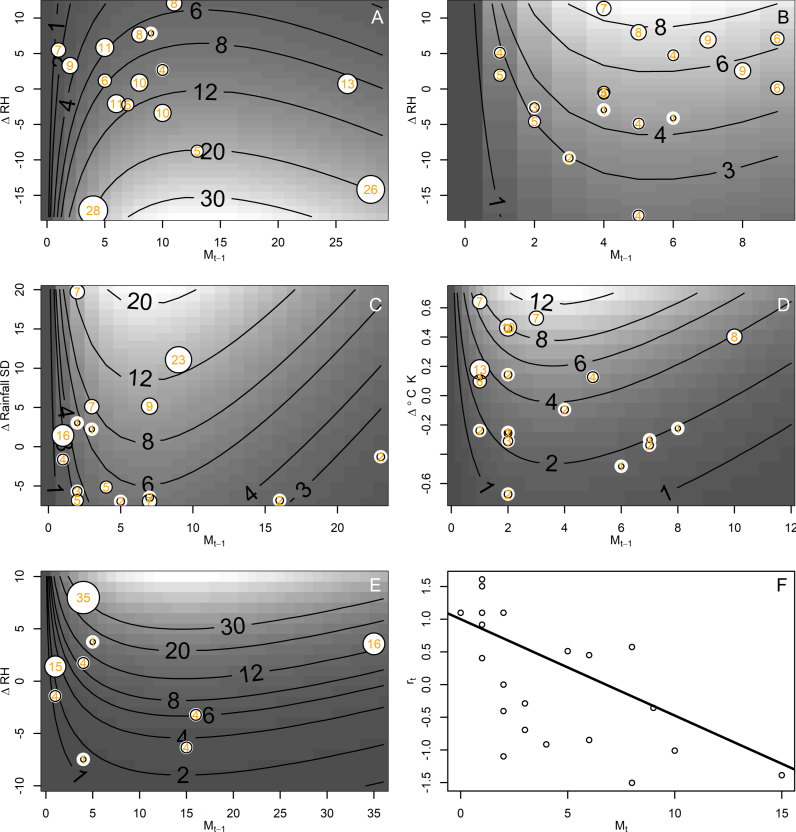
Density dependence. Surfaces of predicted abundance, *M*_*t*_, based on the Ricker model with one climatic covariate for *Aedes japonicus* as a function of relative humidity (**A**), *Aedes triseriatus* as a function of relative humidity (**B**), *Aedes vexans* as a function of the SD of rainfall (**C**), *Anopheles punctipennis* as a function of the kurtosis of temperature (**D**), *Coquillettidia perturbans* as a function of relative humidity (**E**). The expected abundance in panels **A-E** is indicated by isoclines, while observed values are indicated by white circles whose size is proportional to the number of observed mosquitoes, and the observed number of mosquitoes (in orange) is indicated over these circles. The symbol Δ in panels **A**–**E** indicates that the weather covariates were considered as deviations from the mean, i.e. that the mean for the study period was subtracted. **F** Unlike the other mosquito species *Culex pipiens* was not associated with any weather variable, so we present its per capita growth rate (*r*_*t*_ = log(*M*_*t*_) – log(*M*_*t-1*_)) as a function of its density (*M*_*t-1*_).

A graphical representation of best model fit can be observed in Fig. 3, where in general most models satisfactorily reproduce the population dynamics for each of the studied species. For example, *Ae. japonicus* abundance decreased as relative humidity increased during the studied season (Fig. 3A). *Aedes triseriatus* abundance increased as relative humidity increased (Fig. 3B). It was found that *Ae. vexans* abundance increased as the standard deviation of rainfall increased although the numbers observed in the field did not perfectly align, they still followed the predicted trends (Fig. 3C). *Anopheles punctipennis* increased as the kurtosis of temperature increased (Fig. 3D). *Coquillettidia perturbans* increased as relative humidity increased (Fig. 3E). *Culex pipiens* growth rate decreased as the density increased according to Fig. 3F with data points from field observations being scattered around the estimated line. All of these mosquitoes show a clear density dependence in their population dynamics, while most of them, *Cx. pipiens* being the only exception, were sensitive to environmental factors.

## 4 Discussion

This study explored how various weather variables influence the population dynamics of six common mosquito species in Bloomington, Indiana. By applying the Ricker model - a widely used mathematical model for studying population dynamics - we aimed to identify which environmental factors most affected their temporal abundance patterns, and the interaction of such environmental impacts with density-dependent regulation. Our results highlight the diversity in the response to weather changes by mosquito species co-occurring in the same landscape, reflecting both their natural history and response to changing environments and the ubiquity of density-dependent regulation across the species that we studied.

Although density dependence was clearly present across all species we studied, extending observations from other mosquito species and from different environments (Yang et al., 2008a, 2008b; Chaves, 2016; Chaves et al., 2015; Hoshi et al., 2017; Chaves and Moji, 2018), there were differences in the species-specific response to the weather variables. First, *Cx. pipiens* population dynamics was not associated with any of the weather variables studied. This is a surprising result given that time series of this species have been associated with weather variables in several locations from the USA, including similar latitudes in Midwest USA (Hayes, 1975; Hayes and Hsi, 1975; Hayes and Downs, 1980; Gardner et al., 2012, 2013; Hamer et al., 2014), and more subtropical regions in the Southeast USA (Nguyen et al., 2012), Texas (Poh et al., 2019) and California (Barker et al., 2010; Reisen et al., 2010), but also in Taiwan (Ng et al., 2018). We think that one possible explanation for this observation is that the activity of this species as adult is associated with temperature, but primarily in determining the length of its seasonal activity (Spielman, 2001), and that its productivity in the environments we collected our samples was nearly constant. It also highlights the limitations of reducing the set of covariates that are tested when exploring the impacts of weather variables on mosquito population dynamics. We cannot rule that other components of the changing environment impacted the population dynamics.

Secondly, not unexpectedly, and giving the use of adult traps baited with lights and a lure in order to reduce potential biases in the sampling (McDermott and Mullens, 2017), relative humidity (RH) was the most significantly associated with abundance changes in *Ae. japonicus*, *Ae. triseriatus*, and *Cq. perturbans*. As noticed by early studies in mosquito biology, both in the field and the laboratory, adult mosquito activity is highly correlated with relative humidity, with low relative humidity, in general, inhibiting flight related activity (Platt et al., 1957, 1958; Rowley and Graham, 1968; Dow and Gerrish, 1970), which is essential for the successful sampling of mosquitoes with adult traps (Bidlingmayer, 1967; Reisen et al., 2000; Chaves et al., 2020). In the case of *Ae. triseriatus* and *Cq. perturbans* the association with relative humidity was positive, which is something expected under the scenario that activity is highest at higher relative humidity. However, for *Ae. japonicus* the relationship was negative, suggesting that relative humidity might act in conjunction with temperature (Brown et al., 2023), as *Ae. japonicus* seems to have most of its activity at colder temperatures, and which is essential to explain its bimodal abundance patterns with peaks in spring and fall (Chaves and Moji, 2018). *Coquillettidia perturbans* showed the typical pattern of an univoltine species, with a unique peak in adult abundance, a pattern previously observed in temperate (Allan et al., 1981; Batzer and Ranta, 1994), but not in subtropical areas where the species is multivoltine (Lounibos and Escher, 1983; Batzer et al., 2020). Of the species associated with relative humidity, *Ae. triseriatus* presents the most interesting patterns, as previous studies have suggested the importance of rainfall for its oviposition (Kitron et al., 1989; Barker et al., 2003a) and temperature for adult activity (Durso and Craig Jr, 1980), studies that did not consider relative humidity as a potential factor influencing *Ae. triseriatus* population dynamics. Beyond what we observed here, the ecology of this species in the latitudinal strip where Bloomington is located is worth further study regarding climate change. As observed in northern Indiana, this mosquito species presents a highly variable seasonality as adult (Durso and Craig Jr, 1980; Sinsko and Craig, 1979, 1981; Szumlas et al., 1996) which can be related to responses both in behavioral activity but also on the overwintering strategy in response to warming temperatures, for example, by increasing the frequency of larval diapause in relation to egg diapause (Shroyer and Craig, 1980, 1983; Sims, 1982). Moreover, the impacts that warming temperatures might have on the complex microbial ecology supporting the larval ecology of this mosquito (Walker et al., 1991; Kaufman and Walker, 2006; Kaufman et al., 2008; Xu et al., 2008; Norman and Walker, 2017), and the consequences that such changes might have for the transmission of LaCrosse encephalitis virus under climate change are worth studying (Grimstad and Walker, 1991; Day et al., 2023).

For *Ae. vexans*, the critical weather variable was not simply the average amount of rainfall but rather the variability in rainfall patterns, measured by its standard deviation. This species is known to utilize transient water sources, such as temporary pools formed by rainfall. Its positive association with rainfall variability suggests that *Ae. vexans* is well adapted at responding to rainfall events, which create ideal breeding sites. This opportunistic strategy allows it to thrive in environments where water availability fluctuates, making it likely to become more prevalent as extreme weather events become more frequent due to climate change (Chuang et al., 2012a, Chuang et al., 2012b).

The population dynamics of *An. punctipennis* was primarily influenced by the kurtosis of temperature, which reflects the frequency of extreme temperature deviations rather than average conditions. This sensitivity to extreme temperatures rather than gradual changes indicates that *An. punctipennis* is more affected by sudden temperature variation, which might influence its reproductive cycles and survival, or their behavior as adults of *An. punctipennis* have been previously described as thermotropic, having activity towards heat sources (Marchand, 1918). This suggests that *An. punctipennis* populations could be prone to mosquito outbreaks, or sudden changes in population abundance, in regions where temperatures are increasingly variable (Chaves et al., 2014; Chaves, 2017a), potentially affecting its role in pathogen transmission as climate extremes become more common.

In understanding mosquito population dynamics we think that it is also necessary to integrate information about aquatic populations, a limitation of this study, as having such information would allow to better understand delays in the response of mosquito populations to weather patterns (Chaves et al., 2012, 2014) and their stage structure in the field, which is of primary importance to weight the use of larval *vs* adult methods of control to reduce mosquito population size (Chaves et al., 2019). Similarly, in the specific context of Bloomington, IN, USA, a larger sampling effort could also provide insights about other landscapes, for example, areas dominated by housing and with other urban features.

## 5 Conclusions

In summary, our study illustrates the varied ways in which mosquito species respond to environmental changes while undergoing density-dependent regulation. Relative humidity, rainfall variability, and temperature extremes each played a distinct role in shaping the population dynamics of different species. These findings underscore the need for a more nuanced understanding of how mosquito populations are influenced by a combination of intrinsic population controls and external environmental factors. As climate change continues to alter weather patterns, integrating field data with predictive models will be essential to better understand mosquito population dynamics and their response to different methods of vector control.

## CRediT authorship contribution statement

**Aidan Patrick Simons:** Conceptualization, Data curation, Investigation, Writing – original draft, Writing – review & editing. **Amanda Lenfestey:** Investigation, Validation, Visualization, Writing – original draft, Writing – review & editing. **Luis Fernando Chaves:** Conceptualization, Investigation, Writing – original draft, Writing – review & editing, Supervision, Resources.

## Ethical approval

Not applicable.

## Data availability

Data supporting the conclusions of this article are included within the article and in Khan et al. (2024).

## Funding

This study was supported by 10.13039/100006733Indiana University and the Sustainability Scholars Program at 10.13039/100006733Indiana University (Integrated Program in the Environment and Environmental Resilience Institute) through a fellowship to Aidan Patrick Simons.

## Declaration of competing interests

The authors declare that they have no known competing financial interests or personal relationships that could have appeared to influence the work reported in this paper.
